# An analysis of national media coverage of a parental leave reform investigating sentiment, semantics and contributors

**DOI:** 10.1038/s41598-023-49356-y

**Published:** 2024-01-16

**Authors:** Arnault-Quentin Vermillet, Sara Engsig Krejberg, Julie Svinth Nielsen, Christine E. Parsons

**Affiliations:** 1 Department of Clinical Medicine, Interacting Minds Center, Aarhus University, Aarhus, Denmark; 2 Present Address: Interacting Minds Center, Building 1483, Nobelparken, 8000 Aarhus C, Denmark

**Keywords:** Psychology, Human behaviour

## Abstract

While paid leave for fathers after the birth of a child has become increasingly available, mothers still take most of the parental leave. A recent European Union (EU) reform addresses the unequal sharing of leave between parents via earmarking of paid, non-shareable leave to each parent. Given that the reform’s success will depend on uptake by families, we analysed Danish national media coverage to understand how journalists were writing about the reform. We assessed the sentiment and semantics of leave reform coverage compared to general news from the same period, also considering the inferred journalist gender and newspaper political orientation. Parental leave reform articles were slightly more emotional than general news, independent of who authored the article, or the newspaper where it was published. We found a robust difference in the semantics of how female journalists wrote about the reform, relative to male journalists, and that female journalists contributed to media coverage at a higher-than-expected rate. The tendency for media coverage to be written with a non-neutral sentiment can be understood in terms of the enduring political tensions over gender equality, the role of the EU and families’ rights to self-organization. That female journalists over-contributed to media coverage is interesting in understanding topic assignments or interest in parental leave.

## Introduction

Parental leave is often structured as a family entitlement that mothers and fathers can share. However, globally, mothers take more leave than fathers, which is a key contributor to labour market inequality between men and women^1–3^. As a result, there has been considerable interest in policy reforms that incentivize parents to share parental leave more equally. In the EU, a central reform has been to mandate earmarked, non-transferable leave for all member countries from 2019. By 2022, all EU member states were required to provide each parent with nine weeks of non-transferable parental leave.

Parental leave policy reforms aimed at increasing fathers’ leave-taking have been much debated because of their potential for impacting child development and parental health, in addition to household incomes and labour market participation. Greater father involvement has been associated with positive effects on child development^4^. Recent research has suggested beneficial effects of fathers’ leave-taking on both paternal and maternal well-being in the transition to parenthood^5^. However, opponents of reforms have argued that earmarking restricts the choices that parents can make^6^. “Use it or lose it” earmarking policies mean that leave is effectively forfeited if fathers do not take it, resulting in less parental leave overall. Moreover, if fathers do take leave, and are the higher earner, as is typical, households have a reduced total income during the leave period.

## Prior reforms have had different effects across countries

Prior policies aimed at changing paternal leave uptake have had varying results. In Iceland, the earmarked leave policy reforms in the early 2000s shifted fathers from taking a 0% share of leave to 33% of the total leave^7^. Across European countries, reforms from the mid-2000s in Germany and the late 1990s in Denmark have been described as the “least effective”^8^. Prior to the introduction of 2 months of father-specific leave in Germany, 3.5% of all fathers took parental leave. While the proportion of fathers taking leave increased post reform, fathers continued to take markedly less leave than mothers, with fathers’ share of all parental leave months taken at just 7.7% in 2012^9^. The Danish introduction of 2 weeks of paternity leave in 1998 was associated with an estimated two days of extra average leave taken by fathers^10^. The subsequent removal of paternal earmarked leave for fathers in 2002 had little impact on the average leave duration taken by Danish fathers, suggesting that the two reforms had only minor effects.

What might explain the differences across countries in the effectiveness of prior policies to encourage leave-taking in fathers? The leave system, its compensation rates and transferability between partners are important variables associated with fathers’ uptake of leave^11^. Iceland’s policy of high compensation relative to salary (75%), combined with a non-transferable three months of paternal leave, are argued to be important in its effectiveness, relative to other countries offering less generous compensation, transferable between parents. Workplace characteristics and cultures are also argued to have a role in leave uptake^12^, along with wider social norms and expectations about caregiving roles e.g.,^13^.

## The Danish paradox

Denmark has a long history of generous family policies—job-protected parental leave and public provision of childcare—and within Denmark, there is a perception that little discrimination occurs based on gender (EU Report 2019)^14^. Despite the country’s family-oriented policies, Danes tend not to support legal measures for achieving gender balance. In an analysis of 27 member countries, Denmark was the country with by far the smallest proportion of respondents (10%) citing legally binding measures as the best way to achieve gender balance in company boards (EU Report 2012, Women in Decision making)^15^. When asked about legal measures to ensure parity between men and women in politics, Denmark had the lowest support (34%) of 25 EU countries^16^.

Given the Danish disinclination to support legal directives aimed at gender equality, the August 2022 implementation of the EU directive on parental care represents a potentially important sociocultural inflection point. Media representations of the parental leave reform provide both a window into trends in attitudes and thoughts over time (see “culturomics”^17^), but also stand to shape public opinion^18^. Whether a parental leave reform will encourage fathers to take leave depends not only on its economic implications for families but also on how it is presented and understood, societally. To investigate the media presentation of the parental leave reform in Denmark, we first used sentiment analysis on a corpus of news articles about the leave reform. We tested whether the sentiment expressed in articles differed based on the gender of the journalist and also the political alignment of the newspaper, a variable that can impact a newspaper’s information dynamics^19^. We compared parental leave reform articles to other news articles published at the same period. Second, we used topic modelling to estimate the most salient partition of the data into two topics, then examined whether it reflected a division between how male and female journalists and left-oriented and right-oriented newspapers wrote about the reform. Finally, we examined who wrote about parental leave, and the publication venue, to understand contributions to media coverage.

## Materials and methods

### Data

Newspaper articles were obtained from the Danish database Infomedia (https://infomedia.org/), which provides electronic access to articles where the copyright is owned by newspaper publishing houses. The most popular Danish print newspapers and online news sources, as previously identified^20^, were selected. The complete list of sources and the search string used are in Appendix 1. We restricted our search to articles published between 1st of January 2019, when a provisional agreement on the EU directive on parental leave was reached, to the 31st of October 2022, after the law change was implemented in Denmark. Using these criteria, we obtained 428 articles. A de-duplicating process, removing articles published from online and print versions, resulted in 316 articles. Finally, we performed a manual screening, removing articles which contained only a news overview (i.e., when an article gave an overview of several news items), an extract, or articles which were not about earmarked parental leave. Our final corpus consisted of 202 articles, and included opinion pieces, editorials, and all other forms of news article.

We then generated a control corpus by gathering 202 comparison articles from the same time period and from the same media sources. Replacing the parental leave specific string, we used the word “i” (Danish “in”, the most common word in Danish newspapers; Dansk Sprognævn & Det Danske Sprog- og Litteraturselskab^21^) to generate a general corpus. 2.232.477 articles matched these parameters, and after removing duplicate articles, 1.577.411 were left. We used a random number generator to select a final 202 articles. The randomly selected control articles were primarily published in right-oriented newspapers (91%). Journalist gender was manually coded based on journalist name. We refer to the comparison dataset as “General News” throughout for clarity. We performed the same cleaning procedure (e.g., de-duplicating, removal of news overviews) for the “General News” control dataset as for the parental leave articles. In a supplementary analysis, we created a control data set matched to the parental leave articles using three variables, date of publication, gender of the journalist and publication outlet (“Matched General News”; see Supplementary Materials).

### Data analysis

#### Sentiment analysis

We used identical cleaning procedures for all articles, using R 4.1.2 (R Core Team, 2022, for a complete list of packages used, see Appendix 2). Irrelevant text was removed (e.g., date of article downloads, journalist names). The articles were divided into sentences, and each sentence assigned a probability of being neutral, positive, and negative, using the model BERT Tone^22^ in Python 3.10^23^. The returned results of BERT Tone were analyzed using Hierarchical Bayesian Modelling in JAGS^24^. The sentiment probability vector of each sentence was drawn from a Dirichlet Distribution whose parameters were dependent on the article the sentence belonged to. Each article’s parameters were themselves drawn from a Dirichlet Distribution whose parameters were dependent on a higher-level grouping of articles (political orientation and gender).

Next, we examined potential sentiment differences comparing (i) articles about parental leave against the “General News” control articles (n = 404; see also the supplementary analysis on the “Matched General News” control dataset) and (ii) articles about parental leave divided into four independent groups based on political orientation of the newspaper (left-orientated and right-oriented) and gender of journalist (male or female name, gender inferred) (n = 118, removing articles where gender of the writer or political alignment of the outlet could not be inferred). We also performed a qualitative coding of the article titles for both the parental reform articles and the “General News” articles (see Supplementary Materials).

#### Topic modelling

Topic modelling was used to examine differences in language patterns between the articles published in left and right-oriented newspapers, and written by male and female journalists, using Latent Dirichlet Allocation (LDA) modelling. LDA is a generative statistical model, whereby a document is considered as a distribution over topics, while a topic is a distribution over words^25^. To limit our analysis to meaningful differences in language use, we lemmatized all tokens and removed stop words^26^. Words occurring more than 300 times were deleted (high frequency words with low information content; there were 18 unique words which represented about 20% of all words in our corpus). Infrequent words used in fewer than 5 articles were also removed. These infrequent words accounted for 6234 unique words, about 80% of all unique words for only 20% of all words in the corpus. By removing highly frequent and highly infrequent words, we could assess the core similarities and differences across articles in the corpus. After cleaning, there were about 17% of unique words remaining, representing about 60% of total words pre-cleaning. In the raw dataset, we had a total of 56,834 words and 7,553 unique words. After cleaning, we had 33,705 words and 1301 unique words.

Typically, the approach in a topic modelling analysis is to find the hyperparameters that maximize coherence scores (i.e., the degree of semantic similarity between high-scoring words in the topic) or produce the most meaningful topics. We deviate from this approach by instead using LDA to estimate the most salient partition of the data (i.e., words in newspaper articles) into two topics. Notably, our model does not include any information regarding the gender of the author or the political orientation of the newspaper. The model estimated the probability of belonging in topic one and topic two for each word and each article (Topic 1, Topic 2). We then pooled the distribution of mean estimates for the probability of each article as belonging to Topic 1 or Topic 2 based on the newspapers’ political orientation and journalist gender (i.e., four groups). If there are meaningful differences between the mean estimates of the different groups, this would indicate semantic differences in how they report on the parental leave reform.

#### Who is writing about parental leave reform?

We also explored the proportions of male and female journalists who authored articles on parental leave, as well as where articles on parental leave were published (left versus right-oriented newspapers), by comparing these proportions to general news article patterns. To this end, we analysed three days of Danish news media articles selected at random between 01/01 2019 and 31/10 2022. For these three days, a search was performed using the database Infomedia, selecting the major national print media (k = 11) as well as major national web sources (k = 14). A JSON response file was obtained from the database and parsed using Python to extract the journalist's name, media source name, etc. and written to an Excel file. For 17/2/2021, a total of 1036 articles were obtained, and de-duplicated using the Excel function, resulting in a total of 924 articles. For 04/10/2021, 129 duplicates were found and removed, leaving 871 unique articles, and a further 19 articles with a publication date of 05/10/2021 were found and removed, leaving a total of 852. For 12/03/2020, a total of 248 duplicate articles were removed leaving 1228. Most articles were attributed to Ritzau, a Danish news agency (n = 596). These articles were excluded from our analyses. We calculated the proportions of female and male journalists, considering only articles with a name attribution. For political orientation proportions, we used the statements on the homepages of the newspapers to categorize three as left-oriented (Politiken, Ekstra Bladet, Information) and five as right-oriented (Berlingske, B.T., Børsen, Jyllands-Posten, Weekendavisen). Newspapers with no specified orientation were not included in this analysis. This dataset will be referred to as the “3-day dataset” throughout.

## Results

### Sentiment analysis

**Figure 1:**
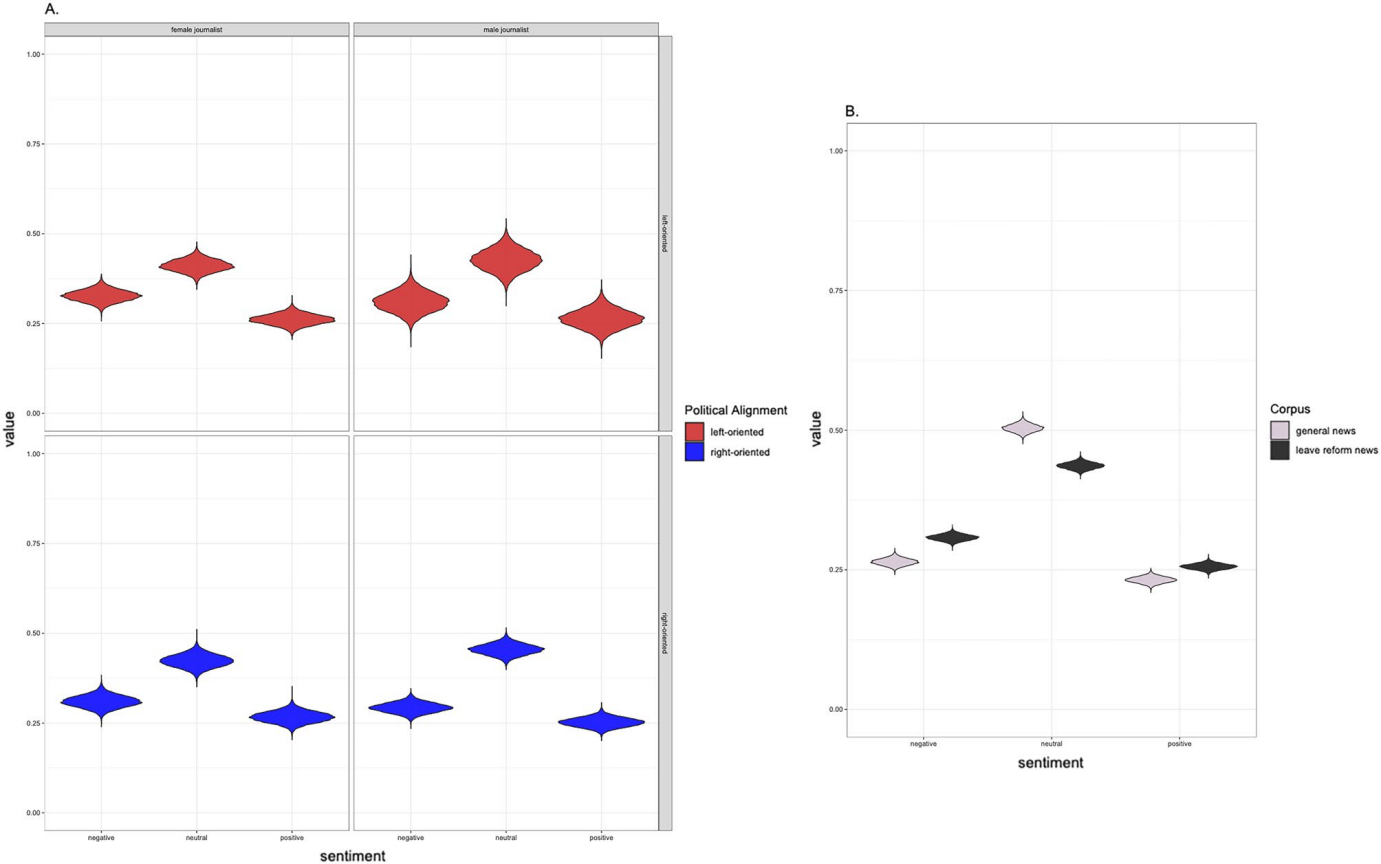
(**A**) The expected value of sentiment across male and female journalists (left panel, right panel) and right and left oriented newspapers (top and bottom panels) in the parental leave reform corpus. Across the four groups, neutral sentences are most prevalent, followed by negative and then positive. The underrepresentation of male journalists writing articles in left-oriented newspapers (17 articles) is illustrated by the greater uncertainty of the estimates. (**B**) The expected value of sentiment in news articles related to the parental leave reform compared to the “General News” control article set.

Overall, a general pattern of neutrality emerged over the news articles. Independent of the gender of the journalist, the political affiliation of the newspaper, or the subject of the article (parental leave reform vs. “General News” control), news articles systematically displayed more neutral sentiment, followed by negative, then positive sentiment (Fig. 1, see also qualitative coding, Supp. Materials). Sentences in the parental leave reform articles were 43.6% (95% CI [42.5–44.8]) likely to be neutral, 30.8% (95% CI [29.7–31.8]) likely to be negative and 25.6% (95% CI [24.6–26.6]) likely to be positive, while in the “General News” control articles, they were 50.4% (95% CI [49–51.8]) likely to be neutral, 26.4% (95% CI [25.3–27.6]) likely to be negative and 23.2% (95% CI [22.1–24.3]) likely to be positive.

Within the parental leave reform corpus, articles written by female journalists in left-oriented newspapers were 41.1% (95% CI [37.9–44.4]) neutral, 32.8% (95% CI [29.7–35.9]) negative, 26.1% (95% CI [23.3–29]) positive. Articles written by female journalists in right-oriented newspapers were 42.3% (95% CI [38.9–45.8]) neutral, 31% (95% CI [27.8–34.3]) negative, and 26.7% (95% CI [23.7–29.7]) positive. Articles written by male journalists in left-oriented newspapers were 42.7% (95% CI [36.9–48.5]) neutral, 31% (95% CI [25.7–36.4]) negative, and 26.3% (95% CI [21.4–31.5]) positive. Articles written by male journalists in right-oriented newspapers were 45.5% (95% CI [42.6–48.5]) neutral, 29.3% (95% CI [26.6–32]) negative, and 25.2% (95% CI [22.8–27.7]) positive.

**Figure 2:**
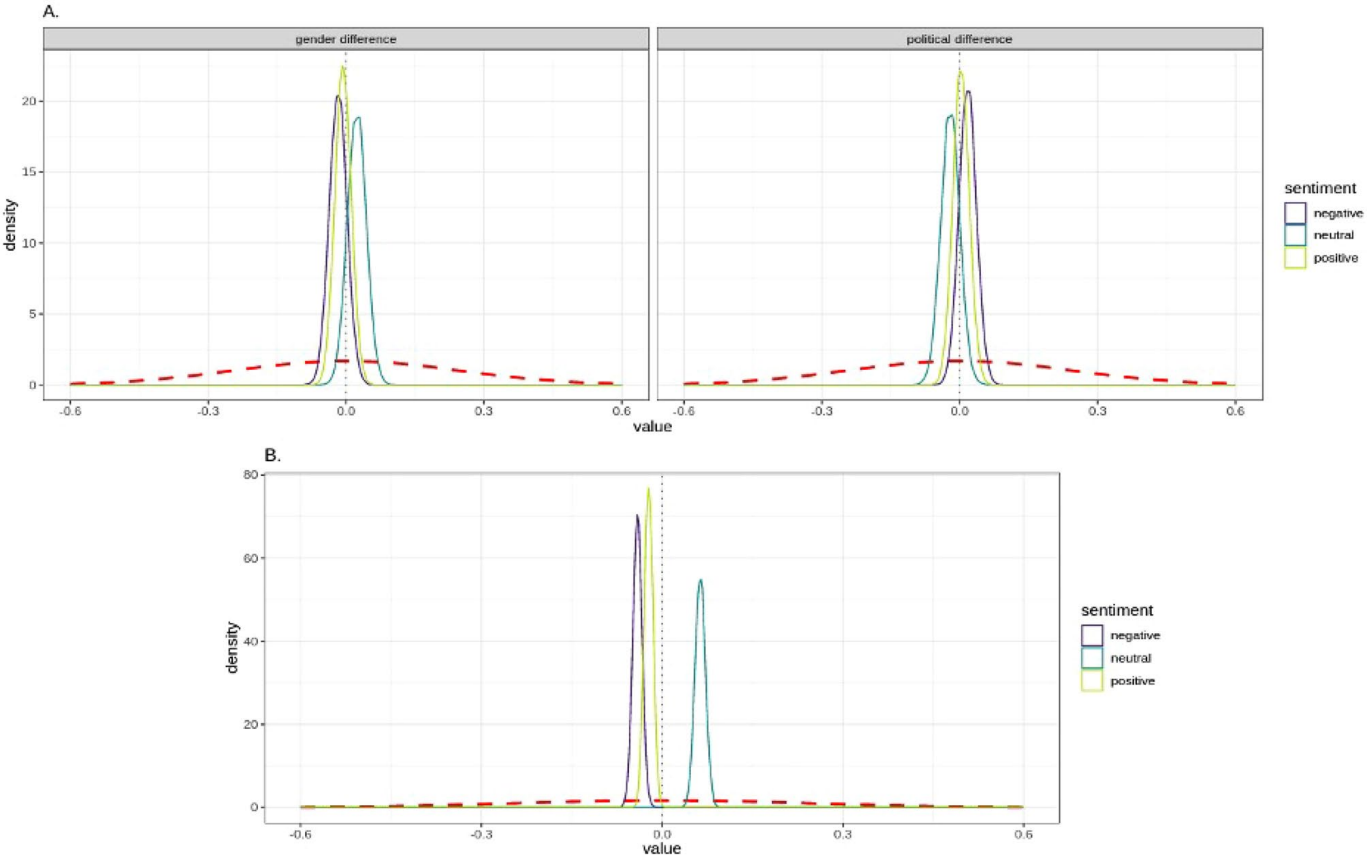
(**A**) The estimated difference in sentiment between male journalists and female journalists (top-left panel) and left-oriented and right-oriented newspapers (top-right panel) in news articles about the parental leave reform. (**B**) The estimated difference in sentiment between articles reporting on parental leave compared with “General News” control articles, independent of journalist gender or political orientation of the newspaper. Values below 0 indicate a higher likelihood for a given sentiment in the parental reform news, while values above 0 indicate a higher likelihood in the “General News”. Across all panels, the red dashed line represents the prior probability of a difference in the model, and the gray dotted line indicates the probability of the absence of difference.

We found no evidence of differences between the sentiment of articles published in the left-oriented and right-oriented newspapers, or between those written by male or female journalists in the leave reform corpus (Fig. 2A, see Supplementary Table 1. for full results). However, our analysis suggested that articles reporting on the parental leave reform were more likely to contain positive or negative sentences and less likely to contain neutral sentences than the “General News” control data (Fig. 2B). “General News” control articles were about 7% more likely to be neutral than parental leave reform articles (ME = 6.7, 95% CI [5.0, 8.6]). Parental leave reform articles were 4% more likely to be negative (ME = -4.4, 95% CI [− 5.9, − 2.8]) and 2% more likely to be positive than “General News” control articles (ME = - 2.4, 95% CI [− 3.9, − 0.9]). The “Matched General News” comparison with parental leave articles showed the same pattern of sentiment difference (see Supplementary Fig. 2). Finally, the manual coding of article titles (see Supplementary Materials) further supported the article sentence sentiment analysis findings, where we found an overrepresentation of positive or negative categorisations for the parental leave articles relative to the “General News” articles.

### Topic modelling

**Figure 3:**
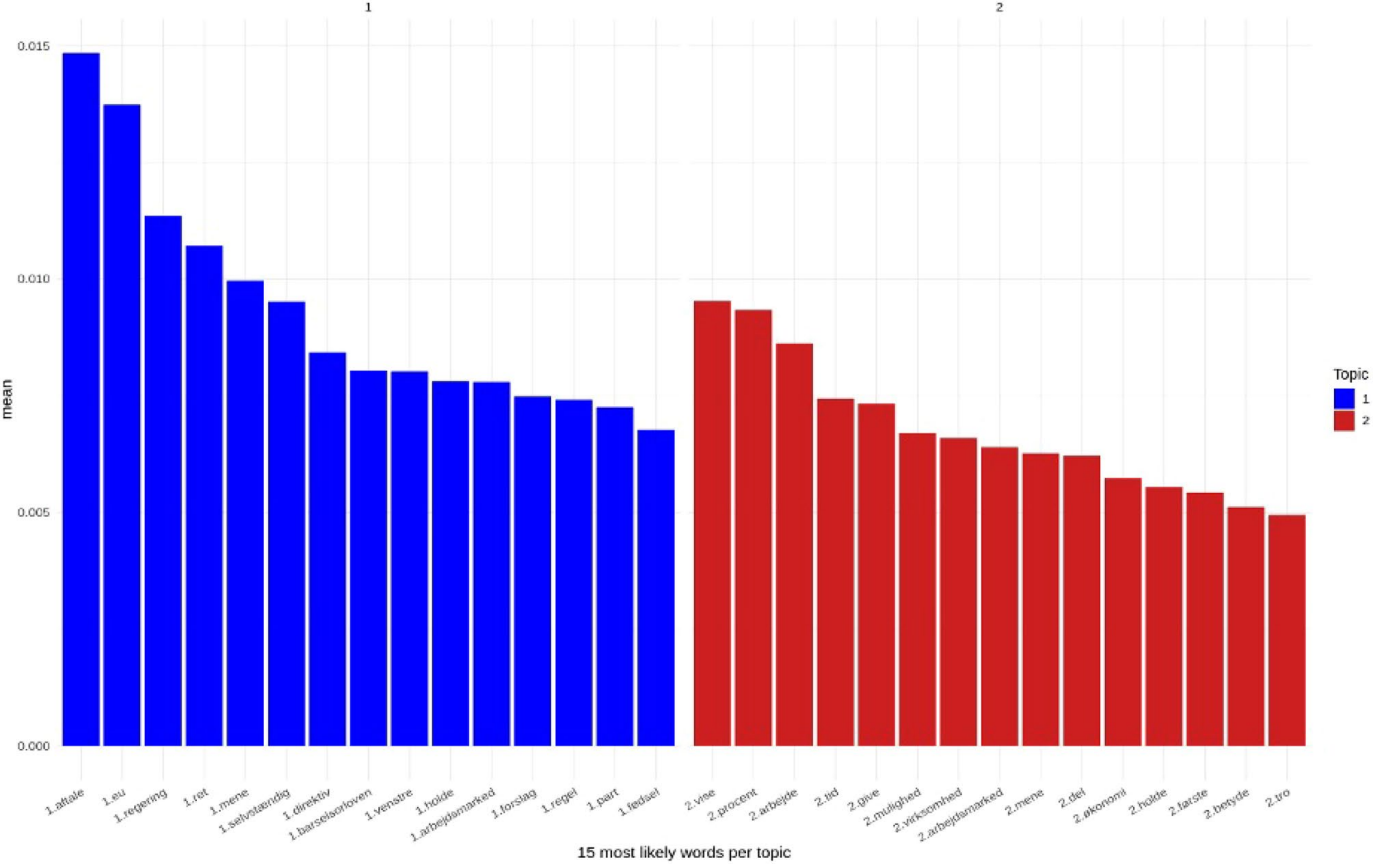
The 15 most likely words in each topic. Translation of keywords: topic 1/blue: a deal, EU, Government, a right (noun), think/believe, independent, directive, parental leave reform, left (noun)/Danish political party, hold/take, labour market, suggestion, rule (noun), part, birth topic 2/red: show, percent, work, time, give, opportunity, company, labor market, think/believe share, economy, hold/take, first, mean/imply, belief.

We investigated whether there was a difference in how right and left-oriented newspapers and male and female journalists wrote about the issue of parental leave using topic modelling. Figure 3 presents the words with the highest probability of appearing in each topic. Some high-likelihood words occurred in both topics (e.g., labour market; hold/take). Topic 1 had several words related to politics (EU, government, directive), whereas Topic 2 had words related to sharing (e.g., time, share, give) and business (e.g., work, company, economy). Fitting the topic model, we estimated the distribution of both topics over all articles (n = 202, see Supplementary Fig. 1). Because there were only 2 topics and probabilities always need to add up to one, the probability distributions of Topics 1 and 2 were opposite and complementary (P(Topic 1) = 1 − P(Topic 2), hence a low probability of Topic 1 was always associated with a high probability of Topic 2). Therefore, we chose to report the results for Topic 2 only.

**Figure 4:**
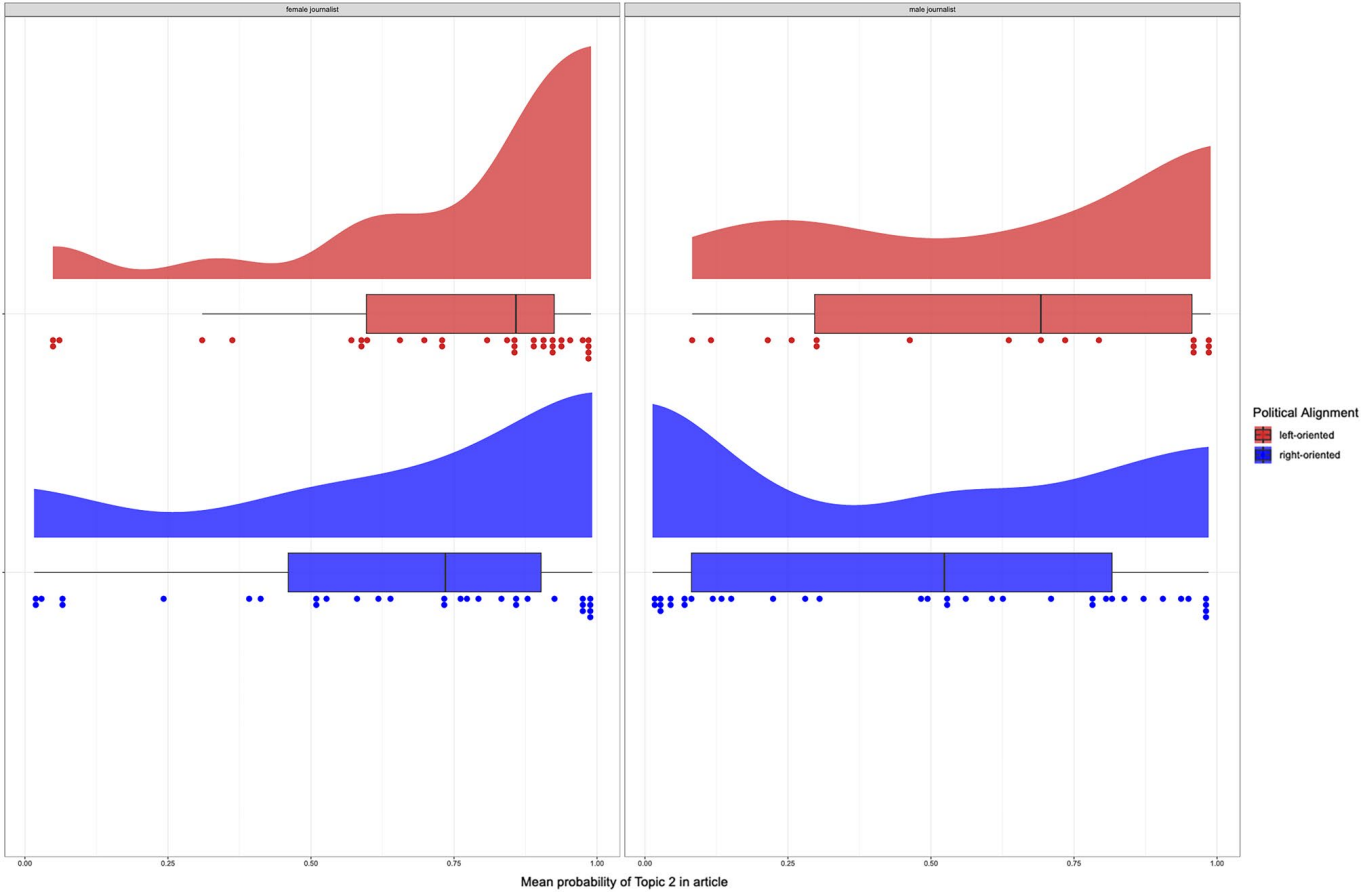
The mean probability of Topic 2 of articles, split by gender and political orientation. Each dot represents one article, while the boxplots and the distributions represent the spread of the estimates across categories. Female journalists included words from Topic 2 (which included words related to time and sharing) in their articles (left panel) more so than male journalists (right panel). For female journalists writing in left-oriented journals, the difference in topic used is particularly pronounced (top left-hand corner).

Figure 4 shows the distributions of mean estimates of probability of Topic 2 in articles, split by the gender of the journalist and the political orientation of the newspaper (articles with no information about gender or political orientation were excluded, n = 118). Articles written by female journalist in left-oriented newspapers were most likely to contain Topic 2 (mean = 0.74, median = 0.86). Articles by female journalists in right-oriented newspapers still favoured the use of Topic 2 words, but to a lesser extent (mean = 0.63, median = 0.74). Estimates for male journalists were generally more spread across both topics, with a mean of 0.61 (median = 0.69) in left-oriented newspapers and 0.48 (median = 0.52) in right-oriented newspapers. While we should be careful in interpreting the results for left-oriented male-written articles because of their small numbers (17), right-oriented male-written articles appeared, at an aggregate level, equally likely to contain both topics.

### Who wrote about parental leave and where were articles published?

Fifty three percent of the articles on parental leave were written by female journalists, compared to 32% of the articles in the “General News” dataset. For the “3-day dataset”, the proportion of articles written by female journalists was between 28 and 33% (see Table 1) and the proportion of articles published in left-oriented newspapers during these days was, on average, between 38 and 50%, compared to 50 to 62% for the right-oriented newspapers. For political orientation, the proportion of articles published in right-oriented newspapers was almost identical for the parental leave reform dataset (56.49%) and the “3-day dataset” (56.73%).

**Table 1:** Proportions of articles authored by female versus male journalists and published in left and right oriented newspapers on the three randomly selected days and for the Parental Leave dataset.

Dataset	Newspaper orientation		Journalist Gender	
	Left-oriented	Right-oriented	Female	Male
12/03-2020	38.30% (n = 370)	61.70% (n = 596)	28.23% (n = 199)	71.77% (n = 506)
17/02-2021	50.46% (n = 330)	49.54% (n = 324)	29.80% (n = 191)	70.20% (n = 450)
04/10-2021	41.05% (n = 259)	58.95% (n = 372)	32.73% (n = 196)	67.28% (n = 403)
Average over the 3 days	43.27%	56.73%	30.25%	69.75%
Parental leave	43.5% (n = 67)	56.49% (n = 87)	53.06% (n = 78)	46.94% (n = 69)

## Discussion

How did the Danish media report on the parental leave reform earmarking additional leave for fathers that came into effect in August 2022? Our sentiment analyses showed that articles about the reform were written with slightly more emotionality than a sample of articles from the “General News” control data, further supported by additional matched control article analyses. We found no differences in article sentiment between left-oriented and right-oriented newspapers, no difference across articles by male and female journalist and no interaction between political orientation and journalist gender. We conclude that articles about the reform were more emotional than we might expect, and this is the case across newspapers of different political orientations and for both male and female journalists. The lack of difference in sentiment between newspapers with different orientations is striking, given that the political parties were almost evenly split in their support of the earmarking of 11 weeks of leave to fathers^27^.

Despite the similarity in emotionality of articles from left-oriented and right-oriented newspapers and male and female journalists, we found marked differences in language semantics. Our topic modelling results indicated a contrast in language use along gender and political lines, noting that journalist gender and newspaper orientation were not entirely independent (i.e., there are more female journalists compared to male journalists writing in left-oriented newspapers compared to right in our “General News” article set). We showed a pronounced difference in the probabilities of the two topics occurring in articles written by female journalists compared to male journalists. The topic-specific difference was especially clear when female journalists wrote articles in left-oriented newspapers (e.g., Politiken). The topic with a high probability of appearing in articles written by female journalists and in left-oriented newspapers included words related to “sharing” and “time”, but also words such as “economy”, “business” and “work”. Our topic model only divided the semantic space in half, finding the two most distinct sets of words that co-occur in news articles. The goal of this broad partition was to investigate the relationship between the most salient semantic difference between articles, and their characteristics (gender and politics). Therefore, more weight should be put on interpreting who uses which topic rather than on interpreting the meaning of the topics themselves.

Female journalists contributed more than might be expected to Danish media articles on parental leave. We found an overrepresentation of female journalists in our dataset of parental-reform related articles, relative to (i) our direct comparison set of articles (“General News”) and (ii) to our analyses of additional randomly selected days of news in Denmark (“3-day dataset”). Female journalists contributed about 53% of the available articles in the parental leave reform data set, but only 30% in the “3-day dataset”. For political orientation, we categorized five newspapers (Berlingske, B.T., Børsen, Jyllands-Posten, and Weekendavisen) as having a political orientation to the right and three as having a political orientation to the left (Politiken, Ekstra Bladet, and Information). The proportion of articles published in right-oriented newspapers was almost identical for the parental leave reform data (56.49%) and the “3-day dataset” (56.73%). We suggest that the issue of parental leave reform is of greater relative focus for female journalists and seems independent of the political orientation of the newspapers.

Female journalists elsewhere have described the phenomenon of not being assigned to cover “hard news” topics such as foreign affairs, crime, and economics, and being assigned to cover topics such as health, children, and education^28,29^. In our control analyses on non-parental reform articles using three days of randomly selected articles, we consistently found that female journalists authored around 30% of the articles. A previous analysis of a day of print media in Denmark reported that 18% of articles were written by female journalists, again further evidence for the minority status of female journalists^30^. Globally, a 2020 report by the Global Media Monitoring Project showed that women reported approximately 40% of stories in traditional news media^31^. The relatively high proportion of female journalist contributions can be understood considering the global proportions of women in media, and the topic of parental leave is often framed as a women’s or social issue. For example, an analysis of local television stories about paid leave in the US showed that where an identifiable individual was included, these were overwhelmingly mothers (91.3%)^32^.

The implementation of the parental leave reform was supported by the major political parties on the left and the centre (e.g., Social Democrats; the Green Left, the Danish Social Liberal Party), along with one major party on the right (Denmark's Liberal Party). The opposition to the leave reforms, as in other countries such as Norway^33^ was from the political right (e.g., Conservative People’s Party). The political right’s opposition centred on the negative consequences of earmarked leave for parental choice, and the potential impact on the child, as well as a disagreement with EU imposition of rules in Denmark^34^. The left’s support of the reform related to achieving gender equality and fathers’ attachment to their children. While the political parties presented different arguments in their discussion of the parental leave reform, we noted only a small effect of newspaper orientation, relative to journalist gender, in our semantic analysis. The newspapers in our analysis report their general political orientation but cannot be considered as perfect mirrors of the stances and views of political parties and actors, as party websites and professional social media accounts might be. An analysis of the semantics of communication directly from political parties, rather than an indirect newspaper categorisation as we have done here, might result in a stronger political orientation effect. Finally, both sets of political arguments on parental leave reform have emotive dimensions, which may account for the lack of sentiment difference between the left and right-oriented newspapers.

Compared to English, we had a more limited range of natural language processing models and tools for Danish^35^. We selected BERT Tone as it showed the best average performance across three sentiment benchmark datasets^36^. BERT Tone has also been used in Danish applied settings, for example, to classify the sentiment of student evaluations in accordance with the students’ numeric scoring of teaching^37^. Although we did find that news regarding the leave reform was less neutral than “General News” articles, the effect was relatively small. We interpret our small effect size, considering the values of neutrality and impartiality that are generally accepted in journalism^38^. It may be that BERT Tone is not the ideal tool to detect sentiment differences in traditional media articles. Alternatively, social media may have been the place where heated or emotive discussions happened. In Norway, for example, it was a social media campaign that catalysed a countermobilization in response to a 2018 parental leave reform^33^. Finally, we analysed news articles from a relatively restricted time period, and future work could compare the difference between control and parental leave articles prior to 2019, with the difference between control and parental leave articles after 2019.

In conclusion, articles on the parental leave reform in Danish media were written with a slightly greater level of emotionality than might be expected, an effect that was consistent across journalist gender and newspaper political orientation. Our topic modelling demonstrated a clear difference in the semantics of how male and female journalists covered the parental leave reform, with smaller effects of the political orientation of the newspaper. Finally, female journalists were robustly over-represented in the article dataset on parental leave reform, in line with other analyses suggesting that women are more likely than men to cover social, family and health issues^39^.

### Supplementary Information

Supplementary Information.

## Data Availability

The coded article data are available in the GitHub folder: https://github.com/Gotticketsto360tour/BarselNews, along with all underlying code to generate the present results. The raw data (i.e., newspaper articles) cannot be stored in a public repository due to copyright constraints. Analyses can, however, be replicated by those with access to Infomedia. For the sentiment analysis, we removed sensitive, identifying information from the data to allow for replication of the analysis.
