# Supplementary material for: An analysis of national media coverage of a parental leave reform investigating sentiment, semantics and contributors
Source: Sci Rep. 2024 Jan 16;14:1407. doi: 10.1038/s41598-023-49356-y (PMC10792055; doi:10.1038/s41598-023-49356-y)
Supplement: Supplementary file 1 — Supplementary Information. [file 41598_2023_49356_MOESM1_ESM.docx]

**Supplementary Materials**

*Qualitative Coding*

Two authors manually coded the titles of the 202 parental leave articles and 202 “General News” articles (CEP, JSN), using 4 categories: positive, negative, neutral, and ambiguous. The ambiguous categories were used for articles where there were both positive and negative expressed emotions, as assessed by the coders. For example, in the title “Holger Rune has reached the point where it's much easier to get better, but also much harder”, both positive sentiment and “easier to get better” and negative sentiment, “much harder” were understood by the coders.

These categories were selected to align with the BERT-based analyses, which examined the articles’ positive, negative, and neutral sentiments. We categorized a higher proportion of the parental leave reform article titles as negative or positive (31% and 18% respectively) compared with the control articles (16% and 10% respectively). A greater proportion of the control articles were categorized as neutral or ambiguous (52% and 22%), relative to the parental leave articles (36% and 15%). Our findings from the manual coding are therefore in broad alignment with the BERT analyses.

We further categorized the parental leave reform article titles as referring to one of the following “advantages/benefits” or “disadvantages/downsides”. Out of the 202 articles, 27 of them were categorized as “advantages/benefits”, which were further divided into subcategories. From these subcategories, most fell into “Improved gender equality” (11) while the second most frequent subcategory was “Benefit to men” (6). The third subcategory included titles focusing on “Benefit to family” (5) while the last two focused on “Economic benefits” (3) and “Father-child bonding” (2).

Thirty-two articles focused on the disadvantages of the parental leave reform in the title, with 10 subcategories being identified. Most of the disadvantages mentioned fell into the category “Negative effect on women” (8), while the second-most addressed was labelled “Harm to the child” (5). Three of the subcategories, labelled as “Right to decide”, “EU interference”, and “Economic harm” were equally represented with 4 articles in each of them. Other subcategories addressed subjects such as “Reduced flexibility” (2) and “Not enough of a change” (2), while the last three titles were coded separately with subcategories labelled “Lose of right to choose” (1), “Less gender equality” (1), and “Earlier to daycare” (1).

Some of the titles included a direct quote, which often was used to set the tone for the opinion expressed in the article. The quotes were categorized as being either positive, ambiguous/neutral, or negative. From these articles, the opinion in the title was most frequently coded as “negative” (43), while 23 of the titles were coded as presenting a positive opinion. Nineteen of the direct quotes were coded as ambiguous/neutral.

**List of resources used**

Appendix 1.

Print newspaper: Politiken, B.T., Jyllands-Posten, Berlingske, Ekstra Bladet, Weekendavisen, Børsen, Børsen lørdag/søndag, Børsen tillæg, Kristeligt-Dagblad and Information articles

Online: dr.dk, tv2.dk, ekstrabladet.dk, ekstrabladet.dk/plus, bt.dk, bt.dk/plus, politiken.dk, B.dk, jp.dk, børsen.dk, information.dk, kristeligt-dagblad.dk and weekendavisen.dk.

To search for articles related to parental leave, we used the following string: [heading, subheading]: ((øremærket OR regler “earmarked” OR “rules”) AND barsel “parental leave”).

Appendix 2. List of software packages used

R: Pdftools (v3.3.3, Ooms (2023)), tidyverse (v1.3.2, Wickham et al., (2019) ), stringr (v1.4.1, Wickham (2022)), tesseract (v5.1.0, Ooms (2022)), tm (v.0.7, Feinerer & Hornik (2023)), stringi( v1.7.12, Gagolewski (2022)), anytime (v0.3.9, Eddelbuettel (2020)), tokenizers (v0.3.0, Mullen et al., (2018)), patchwork, R2jags 0.7-1, gganimate, patchwork, cmdstanr 0.5.2, bayesplot 1.9.0

Python: pandas (v1.5.3, McKinney (2010)), spacy (v3.3.1, Honnibal & Montani (2017)), tqdm (v4.65.0, da Costa-Luis (2019)), danlp (v.0.1.2, Pauli et. al (2021))

Supplementary. Table 1. Results for the sentiment analysis on parental leave reform news articles

|  | | | Mean | SD | Quantile 2.5% | Quantile 97.5% |
| --- | --- | --- | --- | --- | --- | --- |
| Male journalist | Left oriented | positive | 0.263 | 0.026 | 0.214 | 0.314 |
|  |  | neutral | 0.427 | 0.030 | 0.370 | 0.486 |
|  |  | negative | 0.310 | 0.028 | 0.257 | 0.365 |
|  | Right oriented | positive | 0.252 | 0.013 | 0.227 | 0.277 |
|  |  | neutral | 0.455 | 0.015 | 0.425 | 0.485 |
|  |  | negative | 0.293 | 0.014 | 0.266 | 0.320 |
| Female journalist | Left oriented | positive | 0.261 | 0.015 | 0.233 | 0.290 |
|  |  | neutral | 0.412 | 0.017 | 0.379 | 0.444 |
|  |  | negative | 0.327 | 0.016 | 0.296 | 0.358 |
|  | Right oriented | positive | 0.266 | 0.015 | 0.236 | 0.297 |
|  |  | neutral | 0.424 | 0.018 | 0.390 | 0.459 |
|  |  | negative | 0.310 | 0.016 | 0.278 | 0.342 |
|  |  | | Mean | SD | Quantile 2.5% | Quantile 97.5% |
|  | Gender difference | positive | -0.006 | 0.018 | -0.041 | 0.029 |
|  |  | neutral | 0.024 | 0.020 | -0.016 | 0.064 |
|  |  | negative | -0.017 | 0.019 | -0.055 | 0.021 |
|  | Political difference | positive | 0.003 | 0.018 | -0.032 | 0.038 |
|  |  | neutral | -0.020 | 0.021 | -0.061 | 0.020 |
|  |  | negative | 0.017 | 0.019 | -0.019 | 0.055 |


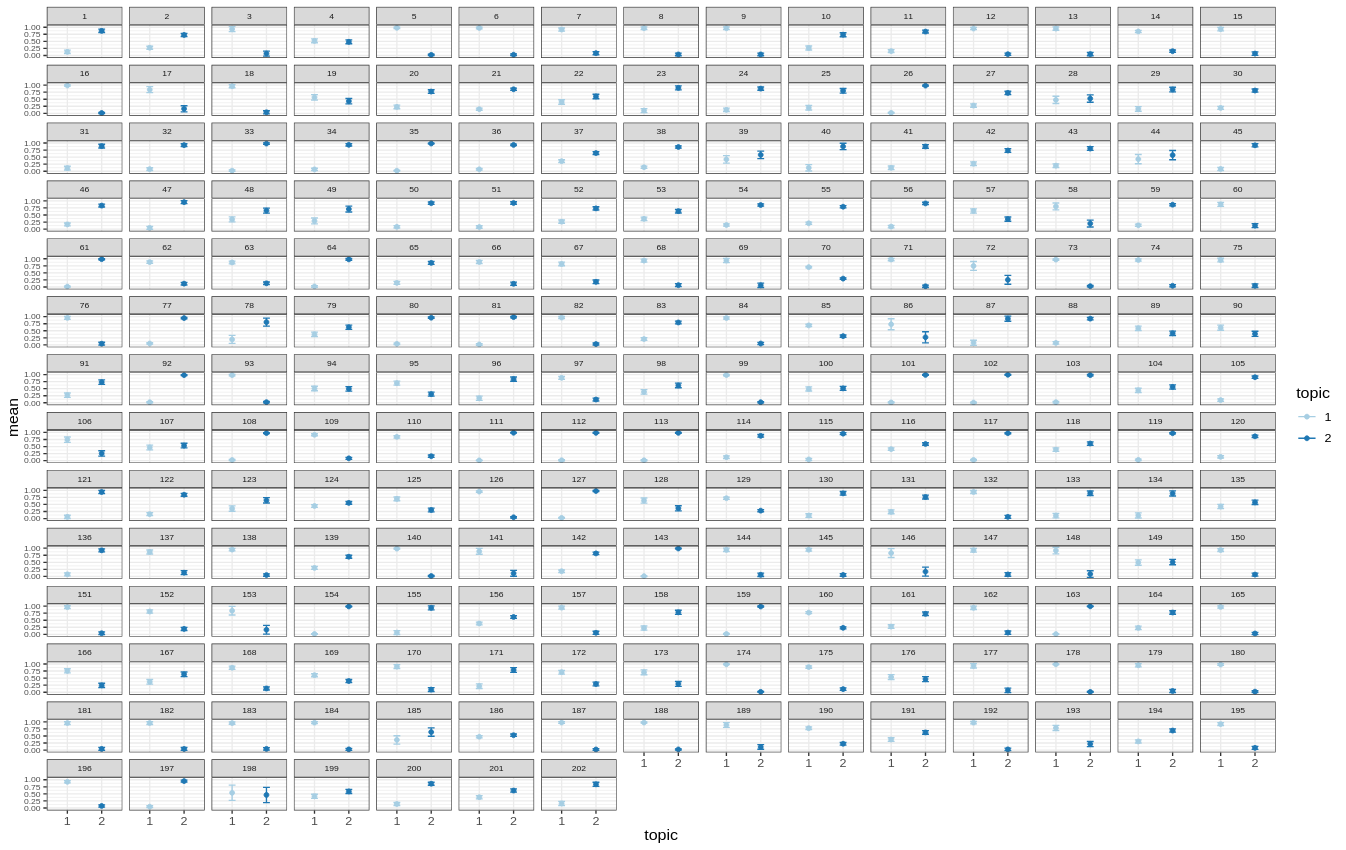

*Supplementary Figure 1.* Mean and Standard Deviation of each topic for each article.

*Comparison using a control dataset matched for outlet, journalist gender and publication day*

The original control dataset for the sentiment analysis was composed of randomly sampled articles from the same newspapers, over the same time period. However, given the distribution of political orientation of outlets, their differing publication rates and the unsupervised nature of the random sampling, the control dataset was composed of a majority of articles from right-oriented outlets (91%). To test if the results of the previous analysis were due to a preponderance of right-oriented outlets, we designed a new control dataset, referred to as “Matched General News”. For each article in the “Leave Reform News” dataset, we extracted control articles about other topics (i.e., not parental leave) from the same newspaper, written on the same day, by a journalist of the same gender. We then randomly selected one article from this subset. We repeated the analysis described in the main article (See Methods: Data Analysis: “Sentiment Analysis”) to compare the sentiments of “Leave Reform News” and “Matched General News”.

Overall, we found the same pattern of effects comparing the parental leave articles to (i) the General News and (ii) the Matched General News. In fact, the difference between Matched General News and the parental leave articles was slightly more pronounced than the difference between the General News and the parental leave articles. In (i), our main sentiment analysis suggested that articles reporting on the parental leave reform were more likely to contain positive or negative sentences and less likely to contain neutral sentences than a set from the “General News” control data chosen randomly over the same period (Figure 2B). Similarly, “Matched General News” control articles were about 9% more likely to be neutral than parental leave reform articles (ME = 9.4, 95% CI [7.8, 11.0]). For (ii), parental leave reform articles were 7% more likely to be negative (ME = -6.5, 95% CI [-7.9, -5.1]) and 3% more likely to be positive than “Matched General News” control articles (ME = -3.0, 95% CI [-4.3, -1.6]) (Supplementary Figure 2B).


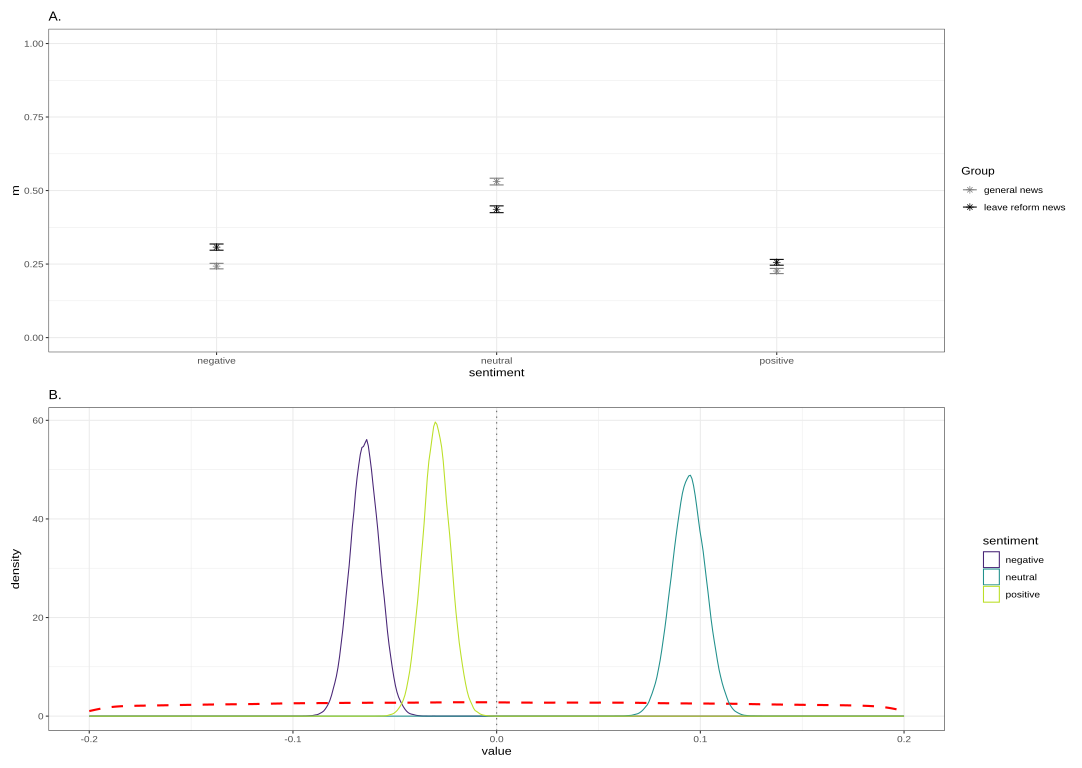


*Supplementary Figure 2.* A. The expected value of sentiment in news articles related to the parental leave reforms compared to the “Matched General News” control article set. The star represents the mean value and error bars the 95% Credible Interval. B. The estimated difference in sentiment between articles reporting on parental leave compared with “Matched General News” control articles, values below 0 indicate a higher likelihood for a given sentiment in the parental reform news, while values above 0 indicate a higher likelihood in the “Matched General News”, independent of journalist gender or political orientation of the newspaper. The red dashed line represents the prior probability of a difference in the model, and the grey dotted line indicates the probability of the absence of difference.
